# Multisensory enhancement elicited by unconscious visual stimuli

**DOI:** 10.1007/s00221-017-5140-z

**Published:** 2017-12-02

**Authors:** Ayla Barutchu, Charles Spence, Glyn W. Humphreys

**Affiliations:** Department of Experimental Psychology, University of Oxford, Oxford, OX1 3UD UK

**Keywords:** Auditory, Visual, Multisensory integration, Consciousness, Posterior Cortical Atrophy, Neurodegeneration

## Abstract

The merging of information from different senses (i.e., multisensory integration) can facilitate information processing. Processing enhancements have been observed with signals that are irrelevant to the task at hand, and with cues that are non-predictive. Such findings are consistent with the notion that multiple sensory signals are sometimes integrated automatically. Multisensory enhancement has even been reported with stimuli that have been presented subliminally, though only with meaningful multisensory relations that have already been learned. The question of whether there exist cases where multisensory effects occur without either learning or awareness has, though, not been clearly established in the literature to date. Here, we present a case study of a patient with Posterior Cortical Atrophy, who was unable to consciously perceive visual stimuli with our task parameters, yet who nevertheless still exhibited signs of multisensory enhancement even with unlearned relations between audiovisual stimuli. In a simple speeded detection task, both response speed, and the variability of reaction times, decreased in a similar manner to controls for multisensory stimuli. These results are consistent with the view that the conscious perception of stimuli and prior learning are not always a prerequisite for multisensory integration to enhance human performance.

## Introduction

Multisensory integration can lead to enhanced performance on many behavioural and cognitive tasks. Perceptual sensitivity (Eramudugolla et al. 2011), accuracy, reaction times [RTs; (e.g., Barutchu et al. 2009; Miller 1982)], and memory and learning (e.g., Alais and Cass 2010; Botta et al. 2011; Fifer et al. 2013; Shams and Seitz 2008) can all be enhanced by multisensory, as compared to unisensory, stimulation. Thus, multisensory integration is potentially important for remedial adaptation and plasticity following brain atrophy, and may provide an important vehicle for neurorehabilitation, particularly for those patients with impaired perceptual awareness. Indeed, in those patients with perceptual deficits, such as hemianopia and neglect, multisensory stimulation can effectively be used to enhance and alter perceptual awareness (e.g., Calamaro et al. 1995; Frassinetti et al. 2005; Leo et al. 2008; Passamonti et al. 2009; Soroker et al. 1995). However, multisensory stimulation can also suppress an observer’s awareness of signals, and have adverse effects on their behavioural performance. For example, in healthy adults, visual stimuli have sometimes been shown to suppress the awareness of simultaneously presented auditory stimuli as in the Colavita visual dominance effect (Sinnett et al. 2008; Spence et al. 2011). However, the Colavita effect is typically only observed on a small proportion of trials when the presentation of a visual stimulus dominates conscious awareness, leading to the extinction of other sensory inputs. At the same time, the extinguished stimulus nevertheless still facilitates visual RTs, even in the absence of awareness (of the auditory input). While other unconscious multisensory effects have been documented for control participants (Cox and Hong 2015; Faivre et al. 2014), it is important to note that they have only been found with previously learned multisensory relations [e.g., in adults, subliminally presented primes of well-learnt stimuli, such as auditory and visual digits can be integrated, but only if the task is first learned using conscious multisensory primes; (Faivre et al. 2014)]. Here, we evaluate a case where visual signals are not perceived, remaining unconscious, and multisensory relations unlearnt.

Multisensory processes are subserved by complex neural networks, involving not only the primary sensory regions, but subcortical regions, such as the superior colliculus and the thalamic nucleus the pulvinar, as well as higher order association regions in frontal, temporal, and parietal brain regions (e.g., Andersen et al. 1997; Driver and Noesselt 2008; Stein and Meredith 1993). Neurons responsive to multiple sensory inputs, and those that modulate their activity as a function of the spatial and temporal proximity of the sensory signals, have been identified in all these brain regions. It is also well-known that both audition and vision have direct projections to both the colliculi and the thalamus, and animal studies in primates and cats have shown that both subcortical regions have independent direct projections not only to primary sensory, but to multisensory association areas, which link to the motor system (e.g., Bignall and Imbert 1969; Cappe et al. 2007, 2009; Grieve et al. 2000; Nelson and Bignall 1973; Stein and Meredith 1993). Thus, based on the structural pathways of the multisensory network, it is reasonable to posit that multisensory signals can be integrated even in the absence of inputs from primary sensory and posterior parietal regions that play an important role in conscious perception (see Koch et al. 2016, for review). Here, we investigate whether multisensory processing in the absence of conscious visual perception due to atrophy to the posterior visual system can indeed enhance motor responses.

In the present study, we used a simple detection task with semantically congruent (i.e., a tweeting bird and a barking dog) and incongruent (i.e., a barking bird or a tweeting dog) presentations of audiovisual stimuli presented at one of a range of auditory-visual stimulus onset asynchronies (SOAs). We predicted multisensory enhancements to be larger for congruent than for incongruent audiovisual stimuli. Unisensory and multisensory processing was assessed with this task in a patient (AB) with Posterior Cortical Atrophy (PCA) and healthy age-matched elderly controls. PCA is a neurodegenerative disorder characterised initially by visual-spatial and visual-perceptual deficits, that, in most cases, include simultanagnosia [i.e., a deficit in simultaneous form perception; (Borruat 2013; Kinsbourne and Warrington 1962)]. AB’s visual perceptual problems were so severe that he had difficulty perceiving individual objects when presented rapidly (i.e., for the durations of less than 1 s—when images were presented for longer durations AB was able to name objects as his language processes and working memory abilities were relatively intact), thus providing us with the ideal rare case with which to assess multisensory processing with unconscious visual signals.

## Materials and methods

### Participants

**Fig. 1:**
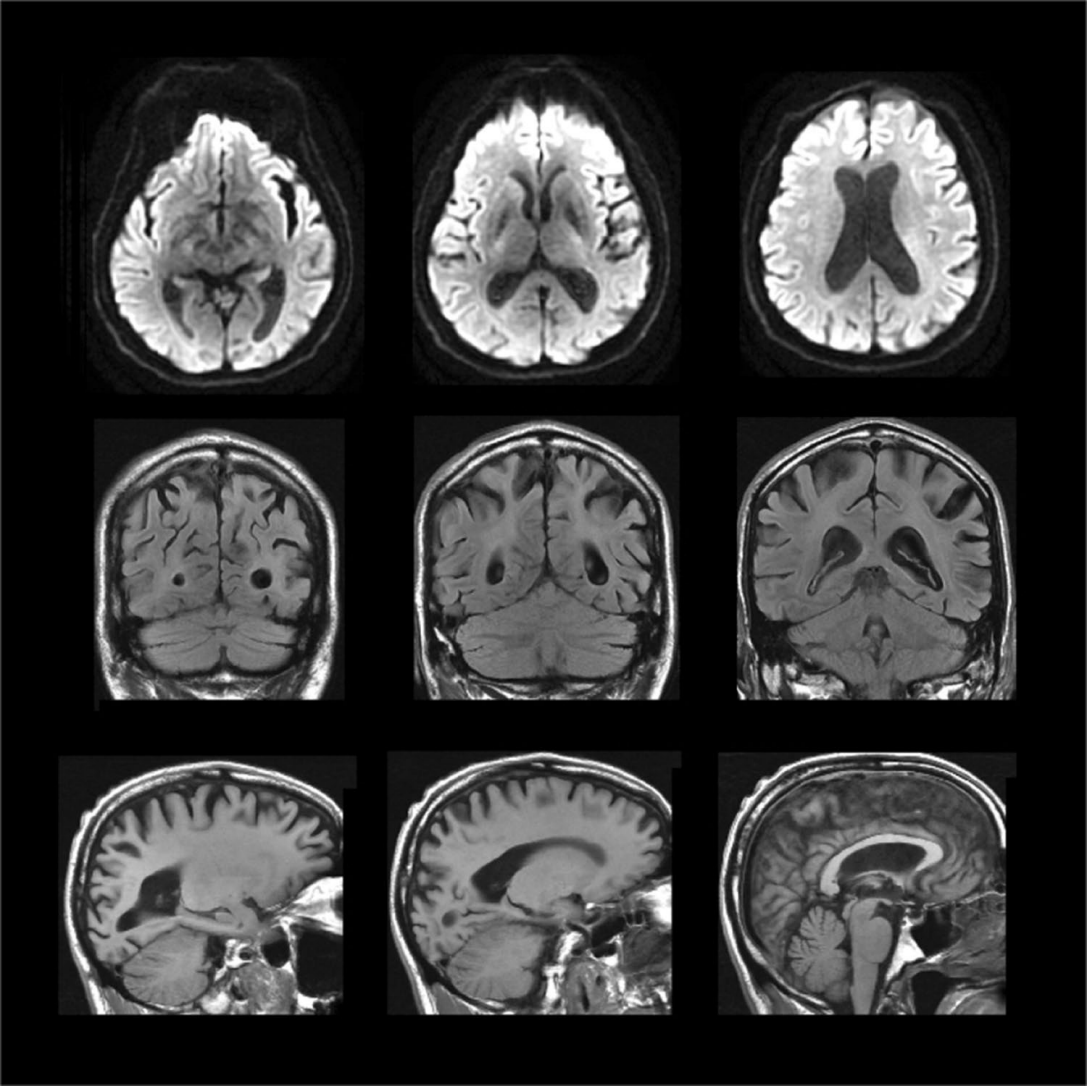
Clinical T1 weighted MRI image of AB’s brain acquired on a 1.5 T scanner showing structural abnormalities and the thinning of the cortex in the posterior of the brain. Ventricular enlargement was also observed

Patient AB, with PCA, was 65 years old at the time of testing (November 2014–February 2015). See Fig. 1 for clinical MRI images. He had profound visuospatial problems and simultanagnosia. For example, he was unable to identify any of the figures in the Boston Cookie Theft picture (Goodglass et al. 2000), except for the woman at the sink. There was also strong evidence of extinction. Given 200 ms exposures of letters 3 degrees to the left or right of his fixation he was able to identify 75% of single items (*N* = 48, 83% left and 67% right) but failed to identify both letters on any of the trials when 2 letters were presented simultaneously (identifying the left item on only 58% of the trials and the right letter on only 25%). Control participants can identify both items under such conditions. There was moderate optic ataxia when reaching for items in his right visual field. He scored 50/100 on the Addenbrookes Cognitive Examination-III (Hsieh et al. 2013) when tested in March 2015 (attention 6/18; memory 16/26; fluency 9/14; language 18/26; visuospatial 1/16). He identified 75% of correctly coloured photographs of common objects but only 44% of line drawings of the same items (*N* = 32). He had an auditory forward digit span of 5, which is within the normal range, and a backwards span of 2.

We tested twelve healthy elderly controls (HC), ranging between 63 and 82 years of age, with no prior history of neurological or psychiatric disorders, normal or corrected to normal vision, and normal hearing (*M* age = 72 years, SD = 5.7 years, 6 males).

All participants provided informed consent prior to participation, and all procedures were ethically approved and strictly adhered to the guidelines of the University of Oxford Central University Research Ethics Committee.

### Stimuli and procedure

All of the participants were presented with auditory (A), visual (V), and audiovisual (AV) stimuli of a dog and a bird. The auditory stimuli were presented from two loudspeakers positioned one on each side of the screen. The stimuli on each side had equal intensity measuring approximately 75 dB at the participant’s ears (note that this set-up led to the sound appearing to come from the centre of the screen). The visual stimuli consisted of coloured pictures of a dog and a bird of approximately 3° of visual angle presented against a white background. The visual stimuli were presented on a 15 inch monitor at a distance of approximately 70 cm at participants’ central point of fixation. For the audiovisual stimuli, semantically congruent (e.g., the image and sound of a dog or a bird), and incongruent (e.g., image of a dog with the sound of a bird or vice versa) stimuli were presented with a variable stimulus onset asynchrony (SOA) of: − 300, − 200, − 100, − 50, 0, 50, 100, 200, and 300 ms (negative SOA values indicates that the auditory stimulus was presented first) for a duration of 380 ms, all presented randomly and with equal probability. Initially, a small fixation cross was presented (0.5° visual angle) for 500 ms duration followed by a random sequence of unisensory and multisensory stimuli with an inter-stimulus interval (ISI) randomly varying between 2000 and 3000 ms in 20 blocks of 44 stimulus presentations, each block lasting approximately 2 min. The participants were instructed to make a simple speeded detection response as rapidly and accurately as possible upon the detection of a stimulus (i.e., on unisensory and multisensory trials alike), and were allowed up to 44 practice trials before beginning the task. The participants were given the option of breaks between each block, and to complete the blocks in 1 session with a 10–15 min break between each set of 10 blocks, or in two sessions (i.e., 10 blocks in each session) on separate days. Participant AB completed the blocks in two sessions, and an additional third set of 10 block in a third session that included blank stimuli that did not require a response to assess his false alarm rates in general (note that the pattern of results did not differ in this additional third session, therefore, AB’s data from all 3 sessions were combined).

### Data processing

For each individual and stimulus condition response, both the RT and accuracy were measured. For multisensory stimuli, RTs were always measured from the onset of the first sensory signal. Only RTs over 100 ms and less than 3 SD of the mean were accepted as correct responses and included in the RT analysis. Less than 2% of RTs were rejected based on this criterion. For each stimulus type, the percentage error rate and mean RTs were calculated. To assess the variability of RT, the coefficient of variation (Cv) of RTs was also calculated by dividing the standard deviation (∂) of the RTs with the mean RT (*µ*) (i.e., Cv = ∂/*µ*).

Multisensory enhancement was assessed by subtracting the RTs for multisensory signals from the faster of the unisensory RTs (note that a positive value depicts faster RTs, while a negative value represents slower RTs for multisensory as compared to unisensory signals).

## Results

### Multisensory enhancement in elderly adults

**Fig. 2:**
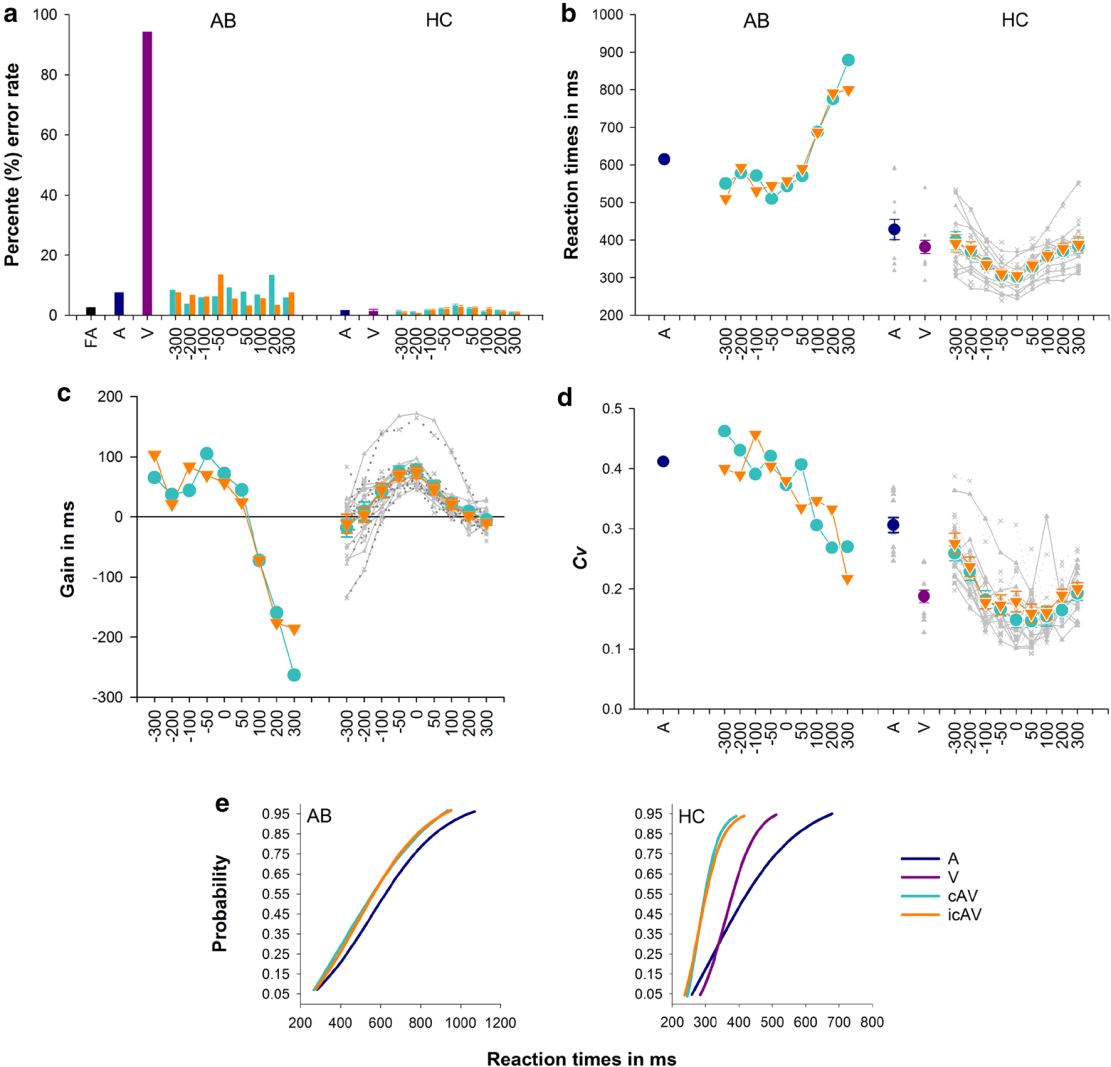
Unisensory and multisensory responses for healthy control participants (HC) and patient AB. **a** Percentage error rates (%) (± SEM for HC) for participant AB and healthy controls (HC) for unisensory stimuli (A—navy bars and V—magenta bars) and multisensory congruent (cAV—cyan bars) and incongruent (icAV—orange bars) stimuli, and stimulus onset asynchronies (SOAs) as shown along the *x*-axis in ms (note for all relevant panels: − SOAs = auditory stimulus presented first and + SOA = visual stimulus presented first). Also for participant AB the black bar = false alarm rates (FA) for no stimulus conditions. **b** Reaction times (RTs) (± SEM for HC) for unisensory and audiovisual stimuli. **c** RTs gain measures (± SEM) for audiovisual stimuli calculated by subtracting the audiovisual stimulus from the faster of the unisensory stimulus (note that positive values depict gain or faster RTs while negative values depict slower RTs for multisensory stimuli). **d** Coefficient of variation (Cv) for unisensory and audiovisual RT measures. Note that for panels **b**, **c**, and **d** grey lines denote individual HC cases (triangles = cAV and  diagnol crosses = icAV stimuli). **e** Cumulative distribution functions for auditory (A), visual (V) and audiovisual congruent (cAV) and incongruent (icAV) stimuli with a 0 SOA for AB and HC participants. Note that AB failed to respond to the majority of visual alone stimuli; for AB a visual CDF cannot be calculated. Note that colour key applies for all panels

Multisensory integration enhanced the speed and reliability of RTs for multisensory stimuli with SOAs less of than 100 ms (Fig. 2). A 2(stimulus type: congruent vs incongruent stimuli) × 9(SOA) repeated measures ANOVA comparing gain measures revealed only a significant main effect of SOA, *F*(8,88) = 23.47, *p* < 0.001, eta = 0.68. Healthy controls exhibited the typical inverse U-shape pattern of responses with optimal benefits (i.e., significantly faster RTs for multisensory stimuli than for unisensory stimuli) observed for audiovisual stimuli in close temporal proximity—i.e., at SOAs of less than 100 ms (see Fig. 2c). Surprisingly, the same pattern of results was observed for both congruent and incongruent multisensory stimuli. The variability of RTs also decreased, particularly when the auditory and visual signals were synchronous and the visual signal preceded the auditory signal (see Fig. 2d for the coefficient of variations of the RTs): The variability of responses for audiovisual stimuli tended to be the same or lower for multisensory as compared to unisensory stimuli. As can be observed in the cumulative probability distributions of RTs presented in Fig. 2e, the entire RT distributions for congruent and incongruent multisensory stimuli were faster than for the unisensory stimuli.

### Multisensory enhancements in PCA

Patient AB (with PCA), just like the control participants, also **r**esponded to auditory and multisensory stimuli (both congruent and incongruent) with relatively high accuracy (i.e., error rates below 20% for all three stimulus types). However, AB failed to respond to the vast majority of the visual stimuli, with an error rate of well over 90% on the visual only trials (see Fig. 2a). Indeed, AB’s response rate for the visual stimuli was comparable to his false alarm rates to catch trials in which no stimulus was presented (see Fig. 2a), thus implying that he was unable to perceive and respond to the unimodal visual stimuli. For example, when asked to discriminate the bird from the dog (data not presented here) and only respond to the bird, AB’s performance was well below chance for both unisensory visual and incongruent multisensory with visual targets stimuli (for both, discrimination errors, i.e., miss rates, were > 95%). Thus, although AB was able to detect congruent target stimuli using the auditory signal, he was unable to reliably match congruent audiovisual signals, further suggesting that he was responding only to the auditory component of the multisensory stimuli. However, when the images were presented on the screen for longer durations (i.e., until the images were detected and named, which was well over 2 s in all cases), AB was able to discriminate and correctly name visual presentations of the bird and the dog. Error rates for control participant were at floor level, thus violating the assumption of normality. Therefore, nonparametric statistics were used to compare the error rates of AB and the control participants; a series of one sample bootstrapping procedures with 10,000 samples and a confidence interval set at 95% were applied for each condition to compare AB’s error rates with control participants. Error rates were significantly higher for AB than for control participants for all unisensory stimuli and for the multisensory stimuli with a large SOA. However, comparisons failed to reach statistical significance for congruent and incongruent multisensory stimuli presented in close temporal proximity: that is, for audiovisual stimuli where the SOA was smaller than 100 ms, AB’s error rates did not differ significantly from controls (for all *p* > 0.05).

RTs for all stimuli were slower for AB than for the control participants (see Fig. 2b). Despite responding more slowly to all stimuli and failing to respond to over 90% of the unimodal visual stimuli, AB still showed large multisensory RT gains comparable to controls, particularly when the auditory signal was presented first. By contrast, when the visual stimulus was presented first, RTs were slower than when the auditory signal was presented alone (note that RTs are measured from the onset of the first signal, and the observed RT delays are similar to the SOAs between the signals when the visual signal is presented first), thus suggesting that AB was unable to perceive the visual stimulus and was responding solely to the auditory signal (see Figs. 2b, 1c). Despite AB showing similar levels of multisensory gain as the controls, his pattern of multisensory gain as a function of the SOA was notably different. First, the typical bell-shaped curve was not observed for AB. That is, his responses were enhanced even when the auditory signal preceded the visual signal by as much as 300 ms (i.e., with an SOA of − 300 ms). Unlike the control participants, he also exhibited significant RT costs, which paralleled the SOA, when the visual stimulus was presented first, further suggesting that AB was unable to perceive the visual signal, and that he was only responding to the auditory signal. Differences in multisensory gain measures (presented in Fig. 2c) between AB and the control participants were assessed using a series of modified one-sample *t* tests (Crawford et al. 2011). Multisensory gains with auditory stimuli leading by 300 ms approached significance (i.e., failing to reach significance with a Bonferroni correction), and significant RT costs were observed when the visual signal led by 200 and 300 ms for both congruent and incongruent multisensory stimuli (see Table 1 for the outcomes of the *t* test). Furthermore, consistent with the data from the control participants, greater multisensory RT variability was only observed for AB with large SOAs and only when audition was presented first. For both AB and control participants, RT variability decreased for audiovisual stimuli when the visual signal was presented first. This result suggests that not only are RTs faster with multisensory stimuli relative to auditory stimuli, their variability also decreases, suggesting a gain in both the speed and reliability of RTs. Despite the severity of AB’s visual perceptual deficits, he was nevertheless still able to gain from multisensory stimulation, improving the speed and reliability of his RTs.

**Table 1:** One-sample *t* test comparisons for congruent and incongruent audiovisual stimuli, comparing multisensory gains in AB to control participants

SOA	Congruent		Incongruent	
	*t* value	*p* value	*t* value	*p* value
− 300	1.52	0.08	2.05	0.03
− 200	0.50	0.31	− 0.41	0.34
− 100	0.04	0.48	− 0.96	0.18
− 50	0.87	0.20	0.02	0.49
0	− 0.18	0.43	− 0.68	0.26
50	− 0.16	0.44	− 0.63	0.27
100	− 3.11	0.005	− 2.90	0.007
200	− 8.98	< 0.001*	− 8.54	< 0.001*
300	− 11.98	< 0.001*	− 10.91	< 0.001*

For all comparisons *df* = 11

*Denotes a significant *t* test with Bonferroni correction

## Discussion

Multisensory enhancement is a robust phenomenon and is often driven by the task relevance of multisensory signals (Barutchu et al. 2013; Sinnett et al. 2008). The present study further demonstrates that the task-relevance of signals can override prior learned semantic associations to enhance multisensory information processing as all of the participants, including controls, showed enhancements of a similar magnitude with congruent and incongruent multisensory stimuli.

It is not surprising that our patient with PCA did not show enhanced semantic congurency effects given his wide spread posterior brain degeneration, and the fact that he was unable to see the visual signals. However, it was surprising that, in this paradigm, previously learnt semantic multisensory associations could be ignored by the control participants, as they were not relevant to the task at hand. This finding deviates from our expectations based on prior studies that have also used domestic animal stimuli as in the present study and demonstrate experience-dependent multisensory enhancements in speeded discrimination tasks (Molholm et al. 2004). The outcomes of the present study are also inconsistent with those studies that have shown prior multisensory subliminal effects that are dependent on semantic crossmodal learned relations (Cox and Hong 2015; Faivre et al. 2014). This divergence in findings can perhaps be explained by the fact that the present study used a speeded detection paradigm, whereby both auditory and visual signals were targets irrespective of the semantic congruence of the stimuli. In contrast, in those previous studies that have used discrimination based paradigms (Molholm et al. 2004), only semantically congruent multisensory stimuli consist of two targets, while semantically incongruent multisensory stimuli consisted of single targets (e.g., if the target animal is a ‘cat’, then a semantically congruent ‘meowing cat’ consists of two targets, while the semantically incongruent stimuli of ‘meowing lamb’ or a ‘baaing cat’ consist of single targets). Other previous research has also demonstrated optimal multisensory enhancement when attention is directed to multiple senses rather than when focused on a single sensory component of a multisensory signal (Miller 1982, 1986). These findings are also consistent with the results of those studies that have demonstrated that some multisensory processes are invariant with respect to semantic congruence [e.g., in semantic categorization tasks (Shepherdson and Miller 2016) and the extinction-like component of the Colavita visual dominance effect (Koppen et al. 2008)]. Here, we further demonstrate the flexibility of the multisensory system, and how the effect of prior semantic learning on multisensory integration can be vetoed depending on the relevance of stimuli and task demands.

Importantly, we demonstrate for the first time that multisensory processing of both semantically congruent and incongruent stimuli can enhance RTs, and improve the reliability of responses, even in a patient with severe visual perceptual deficits. Thus, this study extends previous research that has shown multisensory facilitation effects with subliminal semantically congruent stimuli in healthy young adults (Cox and Hong 2015; Faivre et al. 2014). It also expands on prior research that has shown multisensory integration can help restore conscious awareness of stimuli in neglect and hemianopia patients. Indeed, this case with PCA is very different from a neglect and hemianopia patient, who would typically perceive centrally presented flashing transient stimuli (particularly in the absence of competing visual stimuli as in the present experimental set-up), and even when signals are presented in their neglected or ‘blind’ side, multisensory integration has been shown to enhance conscious perception (e.g., Calamaro et al. 1995; Frassinetti et al. 2005; Leo et al. 2008; Passamonti et al. 2009; Soroker et al. 1995). In contrast, multisensory integration did not reliably restore conscious perception in the patient we studied with PCA. However, despite our patient failing to respond to visual stimuli, he nevertheless still exhibited improvements in response speed, and a decrease in RT variability, under both congruent and incongruent multisensory conditions. Our patient with PCA may also have a larger temporal window of multisensory integration. Unlike in our healthy controls, enhancements were observed when the auditory signal preceded the visual signal by 300 ms. This difference in enhancement between our patient with PCA and the control group failed to reach significance with a Bonferroni correction, though with a *p* value of 0.03 it is importance to acknowledge the possibility of a Type II error. For the multisensory system to be ecologically valid, it needs to tolerate large spatial and temporal discrepancies given the difference in the speed at which auditory and visual signals travel, and differences in neural processing times. Consistent with the findings in our control group, the likelihood of auditory and visual signals being integrated into a unified percept is greatest when auditory and visual signals are spatially and temporarily aligned within 100 ms (e.g., Lewald and Guski 2003), nevertheless, multisensory stimuli can be integrated into unified objects or events even with temporal disparities up to 800 ms (Wallace et al. 2004b). Interestingly, similar to our case with PCA but not our control participants, using a speeded picture categorization task, Chen and Spence (2013) showed multisensory semantic congruence effects only when the auditory stimulus preceded the visual signal by 240 ms or more. In contrast, with SOAs within 100 ms an inhibitory effect was found irrespective of semantic congruence. Differences in findings between studies could be due to the fact that we used a simple detection rather than an image categorization task. Indeed, the spatial and temporal properties of multisensory integration have a complex relationship dependent not only on the task at hand, but the type of signals being integrated (e.g., novel signals vs. naturalistic images and sounds) (e.g., Chen and Spence 2013, 2017; Spence 2013; Stevenson et al. 2012). In this case with PCA, given that the primary visual and posterior parietal brain regions are affected, a degradation or lack of visual information from these brain regions may have led to the broadening of the temporal integration window for multisensory signals to compensate for the loss of visual information.

On the other hand, when the visual signal preceded the auditory signal, the RTs of our patient with PCA slowed significantly relative to the onset of the first signal (in this case vision). This slowing down of RTs was comparable to the SOA between the auditory and visual signal, thus suggesting that the participants were indeed not perceiving the visual signal and only responding to the auditory signal. In this case, the auditory signal needed to precede the visual signal for multisensory integration to enhance performance. Previous studies have shown that multisensory processes are partly dependent on attention (e.g., Alsius et al. 2005; Talsma et al. 2010), and, therefore, the consciously perceived auditory signal may have needed to precede the subconsciously processed visual signal to capture attention to engage multisensory processes and consequently enhance RTs. Thus, in this case, multisensory processes can enhance information processing even in the absence of conscious awareness of the visual stimuli, but only when the auditory signal is in very close temporal proximity with, or precedes, the visual signal.

The present study only focused on the multisensory enhancement of RTs. Response accuracy was at ceiling for our control participants, and in our patients with PCA the response accuracy was not enhanced by semantic congruency, nor by multisensory integration. However, the semantic congruency of subliminal multisensory signals and conscious learning may be important when it comes to enhancing response accuracy and other multisensory phenomena, which should be investigated by further research. Indeed, some multisensory relations that are likely to be learnt, such as the importance of temporal congruence between multisensory signals, were observed in the present study. However, previous adaptation studies have demonstrated that even environmentally well-established processes, such as the temporal and spatial coding of multisensory stimuli, can be recalibrated in adults (e.g., Gallace et al. 2007; Harrar et al. 2016; Simon et al. 2017; Van der Burg et al. 2015). Our participant with PCA may have also showed some signs of such calibration of his temporal multisensory integration window. Indeed protracted learning and environmental experience is important for establishing multisensory processes throughout development (e.g., Barutchu et al. 2009; Gori et al. 2008; Wallace et al. 2004a), nevertheless, the mature adult system remains adaptable, plastic, and is able to rapidly recalibrate to meet the demands of specific tasks, and perhaps even to compensate for changes in sensory perception.

In our participant with PCA, although posterior cortical regions and functions were impaired, given that motor, language and executive processes were relatively functional, a large component of the multisensory cortical and subcortical network over frontal and temporal brain regions, including subcortical areas such as the superior colliculus and thalamus (Driver and Noesselt 2008; Stein and Meredith 1993; Stein and Stanford 2008), may have remained intact to support the observed multisensory motor enhancement. Indeed both the thalamus and the colliculi receive direct inputs from the auditory and the visual sensory systems, and both brain regions have independent ascending and descending projects, not only to cortical primary sensory areas, but also to higher association regions involved in multisensory processing (e.g., Cappe et al. 2009; Grieve et al. 2000; Stein and Meredith 1993). Therefore, to enhance RTs, sub-conscious visual and auditory signals might be integrated in subcortical regions (e.g., thalamus and superior colliculus), or information could be integrated in higher multisensory association areas, bypassing the primary sensory regions needed for conscious perception, before being relayed to motor regions for faster output. Further research is needed to dissociate between these possibilities, and to develop a better understanding how cortical and subcortical multisensory brain areas contribute to conscious and subconscious perceptual processes.

In conclusion, neither prior learning, nor conscious perception of sensory signals, is necessary for the occurrence of multisensory enhancement in this patient with PCA; visual stimuli that remain unconscious can be integrated with information from other sensory systems (no matter whether it is semantically congruent and incongruent) and by so doing enhance the speed and reliability of RTs. This finding may potentially be important for the development of novel rehabilitation strategies, and calls for further research into the use of multisensory signalling in neurorehabilitation, the benefits of which extend well beyond conscious perception as demonstrated in the present study.

## References

[CR1] Alais D, Cass J (2010). Multisensory perceptual learning of temporal order: audiovisual learning transfers to vision but not audition. PLoS One.

[CR2] Alsius A, Navarra J, Campbell R, Soto-Faraco S (2005). Audiovisual integration of speech falters under high attention demands. Curr Biol.

[CR3] Andersen RA, Snyder LH, Bradley DC, Xing J (1997). Multimodal representation of space in the posterior parietal cortex and its use in planning movements. Annu Rev Neurosci.

[CR4] Barutchu A, Crewther DP, Crewther SG (2009). The race that precedes coactivation: development of multisensory facilitation in children. Dev Sci.

[CR5] Barutchu A, Freestone DR, Innes-Brown H, Crewther DP, Crewther SG (2013). Evidence for enhanced multisensory facilitation with stimulus relevance: an electrophysiological investigation. PLoS One.

[CR6] Bignall KE, Imbert M (1969). Polysensory and cortico-cortical projections to frontal lobe of squirrel and rhesus monkeys. Electroencephalogr Clin Neurophysiol.

[CR7] Borruat FX (2013). Posterior cortical atrophy: review of the recent literature. Curr Neurol Neurosci Rep.

[CR8] Botta F, Santangelo V, Raffone A, Sanabria D, Lupianez J, Belardinelli MO (2011). Multisensory integration affects visuo-spatial working memory. J Exp Psychol Hum Percept Perform.

[CR9] Calamaro N, Soroker N, Myslobodsky MS (1995). False recovery from auditory hemineglect produced by source misattribution of auditory stimuli (the ventriloquist effect). Restor Neurol Neurosci.

[CR10] Cappe C, Morel A, Rouiller EM (2007). Thalamocortical and the dual pattern of corticothalamic projections of the posterior parietal cortex in macaque monkeys. Neuroscience.

[CR11] Cappe C, Morel A, Barone P, Rouiller EM (2009). The thalamocortical projection systems in primate: an anatomical support for multisensory and sensorimotor interplay. Cereb Cortex.

[CR12] Chen YC, Spence C (2013). The time-course of the cross-modal semantic modulation of visual picture processing by naturalistic sounds and spoken words. Multisens Res.

[CR13] Chen YC, Spence C (2017). Dissociating the time courses of the cross-modal semantic priming effects elicited by naturalistic sounds and spoken words. Psychon Bull Rev.

[CR14] Cox D, Hong SW (2015). Semantic-based crossmodal processing during visual suppression. Front Psychol.

[CR15] Crawford JR, Garthwaite PH, Ryan K (2011). Comparing a single case to a control sample: testing for neuropsychological deficits and dissociations in the presence of covariates. Cortex.

[CR16] Driver J, Noesselt T (2008). Multisensory interplay reveals crossmodal influences on ‘sensory-specific’ brain regions, neural responses, and judgments. Neuron.

[CR17] Eramudugolla R, Henderson R, Mattingley JB (2011). Effects of audio-visual integration on the detection of masked speech and non-speech sounds. Brain Cogn.

[CR18] Faivre N, Mudrik L, Schwartz N, Koch C (2014). Multisensory integration in complete unawareness: evidence from audiovisual congruency priming. Psychol Sci.

[CR19] Fifer JM, Barutchu A, Shivdasani MN, Crewther SG (2013). Verbal and novel multisensory associative learning in adults. F1000Res.

[CR20] Frassinetti F, Bolognini N, Bottari D, Bonora A, Ladavas E (2005). Audiovisual integration in patients with visual deficit. J Cogn Neurosci.

[CR21] Gallace A, Auvray M, Spence C (2007). The modulation of haptic line bisection by a visual illusion and optokinetic stimulation. Perception.

[CR22] Goodglass H, Kaplan E, Barresi B (2000). Boston diagnostic aphasia examination—third edition (BDAE-3).

[CR23] Gori M, Del Viva M, Sandini G, Burr DC (2008). Young children do not integrate visual and haptic form information. Curr Biol.

[CR24] Grieve KL, Acuna C, Cudeiro J (2000). The primate pulvinar nuclei: vision and action. Trends Neurosci.

[CR25] Harrar V, Harris LR, Spence C (2016). Multisensory integration is independent of perceived simultaneity. Exp Brain Res.

[CR26] Hsieh S, Schubert S, Hoon C, Mioshi E, Hodges JR (2013). Validation of the addenbrooke’s cognitive examination III in frontotemporal dementia and Alzheimer’s disease. Dement Geriatr Cogn Disord.

[CR27] Kinsbourne M, Warrington EK (1962). A disorder of simultaneous form perception. Brain.

[CR28] Koch C, Massimini M, Boly M, Tononi G (2016). Neural correlates of consciousness: progress and problems. Nat Rev Neurosci.

[CR29] Koppen C, Alsius A, Spence C (2008). Semantic congruency and the Colavita visual dominance effect. Exp Brain Res.

[CR30] Leo F, Bolognini N, Passamonti C, Stein BE, Ladavas E (2008). Cross-modal localization in hemianopia: new insights on multisensory integration. Brain.

[CR31] Lewald J, Guski R (2003). Cross-modal perceptual integration of spatially and temporally disparate auditory and visual stimuli. Brain Res Cogn Brain Res.

[CR32] Miller J (1982). Divided attention: evidence for coactivation with redundant signals. Cogn Psychol.

[CR33] Miller J (1986). Timecourse of coactivation in bimodal divided attention. Percept Psychophys.

[CR34] Molholm S, Ritter W, Javitt DC, Foxe JJ (2004). Multisensory visual-auditory object recognition in humans: a high-density electrical mapping study. Cereb Cortex.

[CR35] Nelson CN, Bignall KE (1973). Interactions of sensory and nonspecific thalamic inputs to cortical polysensory units in the squirrel monkey. Exp Neurol.

[CR36] Passamonti C, Bertini C, Ladavas E (2009). Audio-visual stimulation improves oculomotor patterns in patients with hemianopia. Neuropsychologia.

[CR37] Shams L, Seitz AR (2008). Benefits of multisensory learning. Trends Cogn Sci.

[CR38] Shepherdson P, Miller J (2016). Non-semantic contributions to “semantic” redundancy gain. Q J Exp Psychol.

[CR39] Simon DM, Noel JP, Wallace MT (2017). Event related potentials index rapid recalibration to audiovisual temporal asynchrony. Front Integr Neurosci.

[CR40] Sinnett S, Soto-Faraco S, Spence C (2008). The co-occurrence of multisensory competition and facilitation. Acta Psychol (Amst).

[CR41] Soroker N, Calamaro N, Myslobodsky MS (1995). Ventriloquist effect reinstates responsiveness to auditory stimuli in the ‘ignored’ space in patients with hemispatial neglect. J Clin Exp Neuropsychol.

[CR42] Spence C (2013). Just how important is spatial coincidence to multisensory integration? Evaluating the spatial rule. Ann N Y Acad Sci.

[CR43] Spence C, Parise C, Chen YC, Murray MM, Wallace MT (2011). The Colavita visual dominance effect. The neural bases of multisensory processes. frontiers in neuroscience.

[CR44] Stein BE, Meredith MA (1993). The merging of the senses.

[CR45] Stein BE, Stanford TR (2008). Multisensory integration: current issues from the perspective of the single neuron. Nat Rev Neurosci.

[CR46] Stevenson RA, Fister JK, Barnett ZP, Nidiffer AR, Wallace MT (2012). Interactions between the spatial and temporal stimulus factors that influence multisensory integration in human performance. Exp Brain Res.

[CR47] Talsma D, Senkowski D, Soto-Faraco S, Woldorff MG (2010). The multifaceted interplay between attention and multisensory integration. Trends Cogn Sci.

[CR48] Van der Burg E, Alais D, Cass J (2015). Audiovisual temporal recalibration occurs independently at two different time scales. Sci Rep.

[CR49] Wallace MT, Perrault TJ, Hairston WD, Stein BE (2004). Visual experience is necessary for the development of multisensory integration. J Neurosci.

[CR50] Wallace MT, Roberson GE, Hairston WD, Stein BE, Vaughan JW, Schirillo JA (2004). Unifying multisensory signals across time and space. Exp Brain Res.

